# Changes of serum cortisol during pregnancy and labor initiation: an onsite cross-sectional study

**DOI:** 10.3389/fendo.2024.1379693

**Published:** 2024-05-14

**Authors:** Yujuan Chai, Hairong Wang, Daiyu Tang, Yi Wu, Zhonghao Sun, Yuping Zeng, Binmao Zhang, Ben Niu, Xiaojing Dong

**Affiliations:** ^1^ Department of Biomedical Engineering, School of Medicine, Shenzhen University, Shenzhen, Guangdong, China; ^2^ Greater Bay Area International Institute for Innovation, Shenzhen University, Shenzhen, Guangdong, China; ^3^ Department of Obstetrics and Gynecology, Guizhou Provincial People’s Hospital, Guiyang, Guizhou, China; ^4^ Department of Obstetrics and Gynecology, The Second Affiliated Hospital of Chongqing Medical University, Chongqing, China; ^5^ Department of Management, Shenzhen University, Shenzhen, Guangdong, China

**Keywords:** serum cortisol, threatened labor, labor initiation, point-of-care testing, diagnosis of labor

## Abstract

**Background:**

Increased maternal cortisol secretion has been observed during pregnancy and labor. However, due to the limitations in diagnostic methods, the dynamic change of cortisol during the short period between threatened labor and labor is unknown. In this study, we aim to evaluate the changes in serum cortisol during late pregnancy and full-term labor initiation, verifying if cortisol could serve as a biomarker for the diagnosis of labor initiation from threatened labor.

**Methods:**

This cross-sectional onsite study involved 564 participants of 6 different gestational stages (C: Control; T1: Trimester 1; T3: Trimester 3; E: expectant; TL: threatened labor; L: labor), all patients in the E, TL, and L groups were at full term. The serum cortisol concentration was quantified with a point-of-care test (POCT), and the gestation, age, parity, and BMI of participants were documented. Morning serum cortisol was collected between 8:00 and 10:00 a.m., except for the TL and L group women who were tested upon arrival or during latent labor. With cortisol levels or all five variables, L was distinguished from TL using machine learning algorithms.

**Results:**

Significant elevation of cortisol concentration was observed between T1 and T3, or TL and L group (P< 0.001). Women belonging to the E and TL group showed similar gestation week and cortisol levels. Diagnosis of labor initiation using cortisol levels (cutoff = 21.46 μg/dL) yielded sensitivity, specificity, and AUC of 86.50%, 88.60%, and 0.934. With additional variables, a higher specificity (89.29%) was achieved. The diagnostic accuracy of all methods ranged from 85.93% to 87.90%.

**Conclusion:**

Serum cortisol could serve as a potential biomarker for diagnosis of L form TL. The rapid onsite detection of serum cortisol with POCT could facilitate medical decision-making for admission and special treatments, either as an additional parameter or when other technical platforms are not available.

## Introduction

The progression of human labor varies between individuals, which is associated with complicated physiological changes during pregnancy and labor (1). An important medical decision has to be made when a threatened labor (TL) patient visits the hospital, who might enter the latent phase within several hours, or wait for days before labor initiation. Currently, the evaluation for hospital admission is based on the clinical symptoms and cardiotocography (CTG) (2). However, the contractions and early symptoms might sometimes show inconsistency or only continue for a while, and the equipment might not be available in regions with limited medical resources. Thus, it would be helpful if additional test platforms and parameters could assist the decision-making in this emergency scenario.

In the past decades, efforts have been made to identify effective predictors for labor initiation (3–6). Stress regulatory hormones belong to the hypothalamic-pituitary-adrenal axis (HPA axis), corticotropin-releasing hormone (CRH), adreno-cortico-tropic-hormone (ACTH), and cortisol were all reported to be associated with parturition (7–10). Since the second trimester, CRH secreted from the placenta (pCRH) has increased dramatically (11). Maternal plasma CRH levels were significantly higher in preterm women who subsequently gave birth within 24 hours (12). The downstream ACTH and cortisol demonstrate a similar pattern and all three hormones peak during parturition, making them potential biomarkers for both preterm and term labor (13, 14).

As one of the most abundant hormones that directly regulate stress, metabolism, and fetal development, cortisol has been monitored for the prediction of labor in many clinical trials (15, 16). Prediction of preterm birth during 32-36 weeks’ gestation with plasma cortisol and other factors has been achieved with a decision tree model (17). Through a weekly assessment of saliva cortisol and other hormones, Alonso et al. (8) predicted the probability of spontaneous birth from 37-40 weeks gestation. The accuracy calculated from the random forest (RF) model of the full-term women ranges from 73.33% to 85.71%. Despite the improvement in prediction accuracy, both studies overlooked the gap between the latest time for cortisol evaluation (usually the last week before labor) and labor. It is unknown whether cortisol levels would increase during the expectant phase or after threatened labor with mild contractions and pain. Since the duration of the threatened labor phase varies from 1-2 hours to days, it could also be possible that serum cortisol is boosted within a short period before latent labor. Intriguingly, salivary cortisol concentration increased dramatically through different stages of labor, which was likely a reflection of the consistent physiological stress (18). Thus, we hypothesize that the rise of cortisol levels might be significant during the transfer between the TL and labor initiation stage, which is a vital period for doctors to make medical decisions.

To facilitate the diagnosis of labor initiation upon the arrival of TL patients, a fast and convenient onsite examination is preferred. However, hormone tests in previous clinical experiments were conducted afterward (18, 19). Blood CRH with low circulating concentration was mostly detected with radioimmunoassay, which involves radioactive elements and is time-consuming (17, 20). Measurement of ACTH and cortisol was often achieved with the enzyme or luminescence immunoassay, which requires hours for the detection or expensive equipment (8, 13). These limitations in detection tools also hinder the clinical application of the findings.

In this study, the dynamic change of serum cortisol during late pregnancy, TL, and labor initiation stages was investigated for full-term participants. Following the ordinary practice of the obstetric department, a novel point-of-care testing (POCT) platform was incorporated and enabled the rapid detection of cortisol onsite. To our knowledge, this is the first cross-sectional study that evaluates the dynamic change of serum cortisol between TL and labor initiation with a “come-and-test” study design. For clinical decision-making, the cutoff concentration of serum cortisol was calculated and multiple variables were applied to the machine learning algorithms for diagnosis. A clinical workflow was proposed based on current operations that help to make medical decisions.

## Materials and methods

### Study population and clinical data

This cross-sectional study was conducted in the Second Affiliated Hospital of Chongqing Medical University from April 2021 to April 2022, in accordance with the Declaration of Helsinki (1989), and approved by the institutional review board. Informed and written consent was obtained from all participants.

The two major cohorts included 70 healthy nongravid women from the Medical Center (C: Control) and 494 pregnant women from the Department of Obstetrics (T1: Trimester 1; T3: Trimester 3; E: expectant; TL: threatened labor; L: labor). A convenient sampling method was applied. The participants planning to conduct peripheral blood collection for regular health examination or prenatal examination were invited and informed of the share of serum samples with this study to minimize the risk and simplify the procedure. All serum samples were collected between 8:00 and 10:00 am, except for the participants in the TL and L group, whose blood was collected upon administration or labor initiation.

The inclusion criteria were: (1) healthy women of 20-40 years old; (2) able to perform blood collection at 8:00-10:00 am; (3) with known pregnancy time; (4) singleton. The exclusion criteria were: (1) under glucocorticoid therapy; (2) hypertension or hyperglycemia before pregnancy; (3) with known gynecological diseases; (3) with any pregnancy-induced complications that require intervention; (4) planning for a cesarean section.

Demographic information including age, week of pregnancy, parity, and BMI before pregnancy was collected with the permission of participants. The length of pregnancy was confirmed by the time of the latest menstrual period and the B-ultrasound during T1. All patients in the E, TL, and L groups were at full term. Group E was defined as full-term without symptoms. In this study, TL condition was defined as irregular uterine contractions and sensing of fetal descent or bleeding. This group included full-term patients who visited the hospital with signs of labor but entered into the latent phase at various speeds. This situation was similar to the TL in preterm, with the immediate outcome unclear. Labor initiation was marked by regular and gradually increasing uterine contraction lasting ≥ 30 seconds, intermit for 5-6 minutes, accompanied by the progressive disappearance of the cervical canal, dilated cervix, and descent of fetus. The labor status and labor time of participants were verified by partogram.

### Point-of-care detection of serum cortisol

The collection of serum cortisol was conducted at the Medical Center (control) or Department of Obstetrics (pregnant). The coagulation tube was centrifuged at 3500 rpm for 5 min or settled for more than 10 min. Serum cortisol was either measured immediately or stored at 4°C before being tested within 8 h. A POCT platform (Jiangsu NepQD Biotech Ltd. China) for serum cortisol was adopted. A serum sample of 20 μl was mixed with 50 μl diluent, and 60 μl of the mixture was applied to the lateral flow test card. The result of the assay was reported by a portable immunofluorescence analyzer after 10 min incubation at room temperature (NepQD-Infinity-V1, Jiangsu NepQD Biotech Ltd. China). The cortisol test platform has a detection range of 1.00–60.00 μg/dL and an intra-/inter-assay coefficient of variation of 15%.

### Statistical analysis

Data from 564 participants were used for statistical analysis using SPSS Statistics 23.0 (IBM Corporation, Armonk, NY, USA), Python (Jupyter Notebook), and R Studio. The effective sample size for each group (α = 0.05, 1-β = 0.8) was estimated based on the method proposed by Wang and Ji (21), and an online calculator for the cross-sectional study was adopted (http://riskcalc.org:3838/samplesize/). Similar results can be obtained through the Gpower software. Based on the criteria and calculation, the sample sizes of all groups are effective enough for the statistical analysis. Our experimental design was a cross-sectional study based on typical patterns of visits by pregnant women, and we estimate the required sample size based on this design. The formula for estimating the required effective sample size is as follows (21):


$$\text{n=}\frac{2\sigma^{2}{\left(z_{crit}+z_{pow}\right)}^{2}}{D^{2}}$$

Where n is the sample size for each group, $\sigma^{2}$ is the variance of either group (assumed to be equal for both groups), and D is the minimal detectable difference between the two means. The $z_{crit}$ and $z_{pow}$ are the standard normal deviation at a level of significance and 1-β power, respectively. The $z_{crit}$ is 1.96 at a 5% level of significance for two-sided tests. The $z_{pow}$ is 0.84 at 80% power and 1.28 at 90% power.

Demographic information including age, gestation, serum cortisol level, parity, and BMI was summarized, and the distribution of each dataset was verified using the Kolmogorov-Smirnov test. A pairwise comparison of variables between groups was conducted with the Kruskal-Wallis test, and the Spearman’s Rank Correlation test was used to verify the relationship between age, BMI, and cortisol level.

The diagnosis of L from TL was first conducted with cortisol levels only. The receiver operating characteristic (ROC) curve, Youden index, and cutoff value were determined between full-term non-labor groups and labor groups (TL VS L or TL+E VS L). The corresponding sensitivity, specificity, positive predictive value (PPV), and negative predictive value (NPV) were calculated for each pair of datasets. Binary logistic regression with the Forward: Likelihood Ratio model was selected for the distinction from threatened labor to labor using cortisol level, age, gestation, parity, and BMI.

To investigate the role of multiple variables (age, gestation, serum cortisol level, parity, and BMI), RF and Support Vector Machine (SVM) models were adopted. A 10-fold cross-validation approach suitable for a limited dataset was used for the performance verification of each machine-learning method, reducing the risk of overfitting and increasing the generalization of models (22). Decision curves and calibration curves were constructed to evaluate the effects of different diagnostic methods. The threshold probability is defined as the minimum probability of a disease or situation requiring further intervention (risk probability). The decision curve was applied to compare the net benefit of the diagnosis at different threshold probabilities with the following equation:


$$\text{Net Benefit=}\frac{\text{True Positive Count}}{n}-\frac{\text{False Positive Count}}{n}*\left(\frac{1-\text{Threshold Probability}}{\text{Threshold Probability}}\right)$$

An alternative expression of the net benefit was also adopted to demonstrate the interventions avoided with the following equation and methodology (23):


Interventions Avoided per 100 Patients = (1- False Positive Rate)∗100

The calibration curve connecting the scatter points of the actual and predicted incidence was used to demonstrate the performance of all models over the entire range of possible predictions.

## Results

### Study population and clinical data

The mean age of all participants ranged from 27.26 to 29.67 and the mean parity ranged from 0.20 to 0.54. Serum cortisol levels in all groups except group L followed a normal distribution (**Table 1**). A significant difference was found in age (P< 0.05), but not in cortisol level, parity, and BMI for group C and T1. Comparison between groups E, TL, and L showed similarities in gestation week, but age differences (P< 0.01) and BMI (P< 0.001). No correlation was identified between serum cortisol level and age or BMI, but age and BMI were positively correlated (P< 0.01).

**Table 1:** Demographic information and serum cortisol concentration of patients in each group.

Groups	C (n = 70)	T1 (n = 70)	T3 (n = 140)	E (n = 70)	TL (n = 140)	L (n = 74)
Age (Y)	27.00(5.00)^*^	30.00(3.75)^*^	28.00(6.00)^*^	29.67 ± 4.31	28.00(5.00)^*^	27.00(6.00)^*^
Gestation (Week)	NA	12.14(0.86)^*^	31.00(3.04)^*^	39.00(1.78)^*^	39.29(1.33)^*^	39.01 ± 0.99
Cortisol (μg/dL)	8.35 ± 1.96	8.26 ± 3.24	14.96 ± 3.20	16.60 ± 3.72	16.57 ± 4.94	26.82(13.52)^*^
Parity	0.20 ± 0.40^*^	0.51 ± 0.70^*^	0.54 ± 0.67^*^	0.40 ± 0.52^*^	0.26 ± 0.47^*^	0.34 ± 0.56^*^
BMI	20.54(2.55)^*^	20.98 ± 1.68	21.02(2.26)^*^	22.28 ± 1.85	21.03 ± 2.01	20.34 ± 2.14

Data following normal distribution are given as mean ± SD, data with non-parametric distribution are presented as median (IQR); data for parity were given as mean ± SD to provide more information despite they are non-parametric. C, Control; T1, Trimester 1; T3, Trimester 3; E, expectant; TL, threatened labor; L, labor. * p<0.05 for Kolmogorov-Smirnov test.

### Change in serum cortisol levels

**Figure 1:**
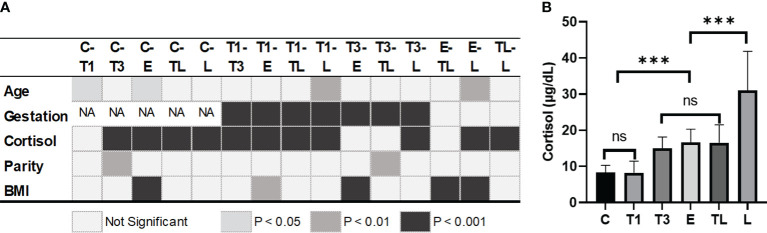
Difference of variables between groups at each significant level **(A)** and cortisol level on different gestation weeks **(B)**. ***: p<0.001; C, Control; T1, Trimester 1; T3, Trimester 3; E, expectant; TL, threatened labor; L, labor; BMI, body mass index before pregnancy. ns, not significant.

The POCT of cortisol revealed no significant difference between group C and T1, or between T3, E, and TL (**Figure 1**). However, a sharp increase was documented in serum cortisol concentration between groups T1 and T3, as well as TL and L, with no differences in age, parity, and BMI (**Figures 1A, B**).

### Diagnosis of labor from threatened labor

**Figure 2:**
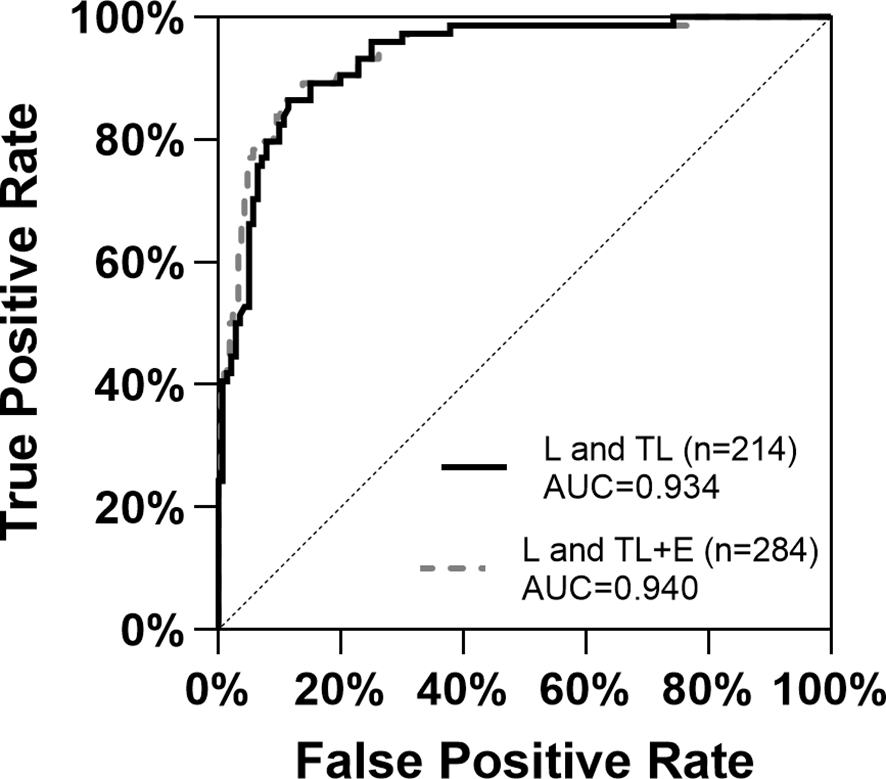
ROC curves for the diagnosis of labor initiation with the two datasets based on serum cortisol levels. E, expectant; TL, threatened labor; L, labor; AUC, Area Under Curve.

117 of the 140 samples in the TL group had an exact time between threatened labor and labor, with a median of 14 hours (IQ1-IQ3 = 7 hours ~ 25 hours). Women with similar gestation weeks from group E, TL, and L were included in the diagnosis of labor initiation. The ROC curves (**Figure 2**) constructed based on maternal serum cortisol levels demonstrated similar shapes and AUCs for the two sets of data: L and TL (n=214) and L and TL+E (n=284). For group L and TL, the Youden index was 0.751, and the cutoff value for cortisol was 21.46 μg/dL (AUC=0.934, CI 95%=0.900-0.967). For group L and TL+E (n=284), the Youden index was 0.760, and the cutoff value was calculated to be 21.62 μg/dL (AUC=0.940, CI 95%=0.909-0.970). Based on cortisol only, and the cutoff value calculated with current data, 124 individuals in the TL group showed lower cortisol levels than the cutoff, which will be diagnosed as true negative (88.57%), and 60 individuals from the L group will be diagnosed as true positive (85.71%).

A binary logistic regression was then adopted to verify the role of multiple variables (**Table 2**). Only cortisol level and age contributed significantly to the model, with the former positively correlated to the labor phase (P = 0.000) and the latter negatively correlated (P = 0.024 and P = 0.008). The result of the logistic regression analysis depended mainly on the serum cortisol level.

**Table 2:** Results of binary logistic regression on the two datasets.

L and TL (n=214)	variable	B	SE	P	OR	95% CI
step1	Cortisol	0.342	0.051	0.000	1.408	1.274-1.556
	constant	-8.043	1.121	0.000	0.000	
step2	Age	-0.140	0.062	0.024	0.869	0.770-0.982
	Cortisol	0.347	0.052	0.000	1.414	1.278-1566
	constant	-4.246	1.924	0.027	0.014	
L and TL+E (n=284)	variable	B	SE	P	OR	95%CI
step1	Cortisol	0.375	0.052	0.000	1.454	1.314-1.610
	constant	-9.096	1.140	0.000	0.000	
step2	Age	-0.159	0.060	0.008	0.853	0.759-0.959
	Cortisol	0.382	0.053	0.000	1.465	1.320-1.625
	constant	-4.820	1.851	0.009	0.008	

Variable entered in step 1: Cortisol; Variable entered in step 2: Age.

To better elucidate the influence of different variables on the diagnosis of labor, two machine learning algorithms, RF and SVM suitable for the small dataset were applied. For L and TL groups, SVM demonstrated slightly better diagnostic power, but for L, TL, and E groups, RF was better for accuracy, sensitivity, and NPV (**Table 3**).

**Table 3:** Performance comparison for the prediction models.

	L and TL (n = 214)				L and TL+E (n = 284)			
	Full dataset		10-fold cross-validation		Full dataset		10-fold cross-validation	
	Cortisol	LR	RF	SVM	Cortisol	LR	RF	SVM
Accuracy	87.90%	87.38%	85.93%	86.41%	88.70%	89.79%	89.63%	88.35%
Sensitivity	86.50%	86.15%	79.11%	80.36%	86.50%	85.71%	76.43%	73.57%
Specificity	88.60%	87.92%	89.29%	89.29%	89.50%	90.95%	91.91%	93.33%
PPV	80.00%	75.68%	80.87%	81.09%	74.40%	72.97%	82.24%	82.67%
NPV	92.50%	93.57%	89.80%	90.34%	94.40%	95.71%	92.06%	91.42%
AUC	0.934	0.939	0.896	0.913	0.940	0.945	0.896	0.931

LR, Logistic Regression; RF, Random Forest; SVM, Support Vector Machine; PPV, Positive Predictive Value; NPV, Negative Predictive Value; AUC, Area Under Curve; ROC, Receiver Operating Characteristic; AUC, Area Under Curve.

### Comparison of diagnostic methods

A parallel comparison of the 4 classification methods suggested that the serum cortisol level should be an effective biomarker for the diagnosis of labor initiation from TL. With the serum cortisol level alone, a good sensitivity of 86.50% could be achieved. The logistic regression produced the highest NPV (93.57% and 95.71%) and AUC (0.939 and 0.945), but the SVM generated the best specificity (89.29% and 93.33%) and PPV (81.09% and 82.67%). For the patients with symptoms (TL and L group), current methods could reach an accuracy of 85.93%-87.90% for the diagnosis of labor initiation.

**Figure 3:**
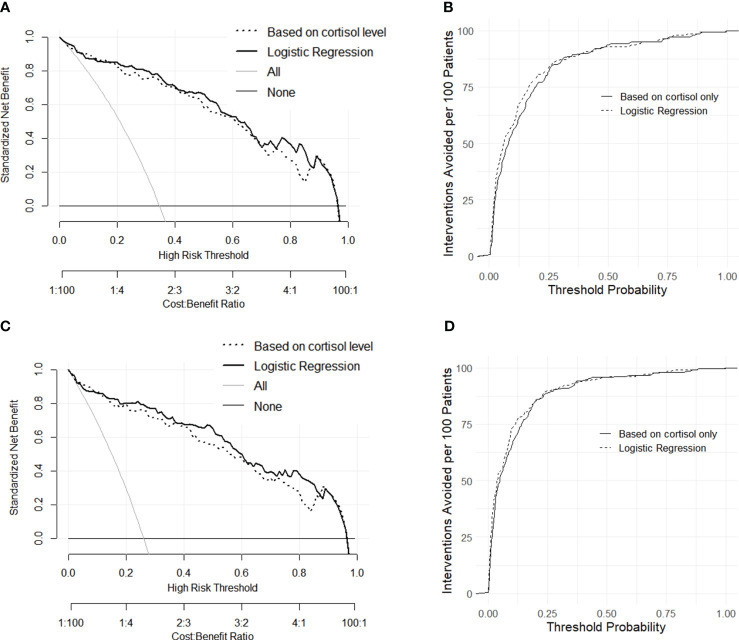
Decision curves of net benefit constructed with dataset L and TL **(A)** and the alternative expression of decision curves as interventions avoided per 100 patients **(B)**. Decision curves of net benefit constructed with dataset L and E+TL **(C)** and the alternative expression of decision curves as interventions avoided per 100 patients **(D)**.

To integrate clinical utility into the analysis and meet the practical needs of decision-making, decision curves were constructed for the comparison of classification methods. As shown in **Figure 3**, the net benefit of the binary logistic regression was similar to that of the prediction based on single indicator cortisol for most of the threshold range, and both approaches demonstrated good utilities for the need. In an alternative expression of the net benefit (**Figures 3B, D**), the interventions avoided (sending all patients with signs of labor for admission and clinical observation) were high for both models, as the two curves were close to each other at all threshold probabilities. This implied that, at a risk threshold of 25%, adoption of either model would be equivalent to a strategy that reduces the number of unnecessary arrangements by roughly 80 per 100 (23).

**Figure 4:**
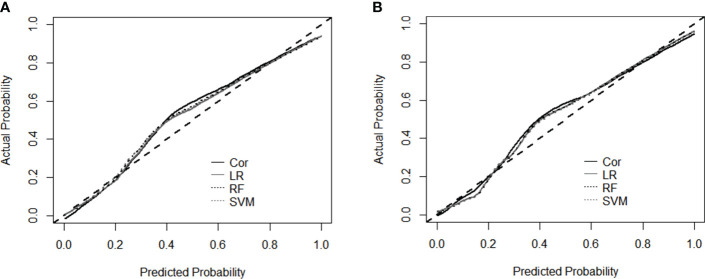
Calibration curves for the performance evaluation of four models using dataset L and TL **(A)**, and dataset L and TL+E **(B)**. Cor, cortisol; LR, Logistic Regression; RF, Random Forest; SVM, Support Vector Machine.

Calibration curves constructed for the four models revealed the residuals of model estimates and prevented model overfitting. For both datasets (**Figures 4A, B**), the shapes of the two traditional methods and two machine learning models supported good diagnosis ability. The similarity of the calibration curves indicated no overfitting of the machine learning models, and the prediction performance should be reliable.

## Discussion

With this onsite cross-sectional study, we found that the morning serum cortisol levels increased gradually during pregnancy. A sharp elevation of serum cortisol concentration was first reported within the relatively short period between TL and the latent phase, which could facilitate the diagnosis of labor initiation (AUC 0.934-0.940). The demographic information of patients might help to further improve the performance of the algorithms.

Different from previous longitudinal studies (8, 24, 25), interpersonal variation is included in this study to mimic the real situation of medical examinations, and the full-term patients were grouped based on physiological changes and symptoms (E, TL, and L) rather than a broad gestation age. Consistent with previous research, our study at the population level also revealed an increment of morning serum cortisol during T1 and T3 (**Table 1**) (25). During late pregnancy (T3 to E), a graduate elevation of morning serum cortisol was observed (14.96-16.60 μg/dL), which was different from the significant increment of salivary cortisol reported by Alonso et al. after week 37 (8). This divergence may be due to the district secretion pattern of different tissues, or the relatively large gestation week variation in the T3 group. Based on these findings, we postulate that the elevation of cortisol secretion during pregnancy should be a common phenomenon in both preterm and full-term women (8, 24).

Interestingly, cortisol levels in the E group were found similar to those in the TL group, although the latter demonstrated typical symptoms and pain. To our knowledge, no previous study has compared the cortisol levels for these two groups. Between the E and TL groups, there was a clear difference in sampling time: blood was drawn at the “high point” of the day for the E group (26, 27), and randomly for the TL group. However, no correlation was identified between the blood sampling time and cortisol level, probably because the neuroendocrine change caused by the stress and pain of TL has already overcome the diurnal variation (24).

Within the short period between TL and L, serum cortisol levels nearly doubled (16.57-31.04 μg/dL), but no disparity in gestation week was found between group L, E, or TL. As verified through correlation tests, age, and BMI were not related to cortisol levels. Therefore, the differences in cortisol levels among the three groups were not likely caused by variations in gestation week, age, or BMI, but caused by the feedforward cycle generated by pCRH (along with ACTH and cortisol) which peaks during parturition (28). There could also be confounding factors that trigger the release of extra cortisol, such as physical or mental stress (pain, fear, or anxiety) and mild endocrine alterations (14, 18). However, our previous study with the same cortisol test identified only a slightly higher level of cortisol in women with mild to severe anxiety (29), suggesting that the major driving force of the extremely high cortisol level should be labor initiation. The labor group of previous studies included preterm patients of different conditions: some showed little symptoms (like our E group), but some with dilation of 2-4 cm (17, 30). In a labor group with different physiological conditions, the sharp increase of cortisol might be overlooked.

To verify the performance of serum cortisol level as a biomarker for labor initiation, ROC curves were constructed for groups TL and L, as well as E+TL and L (**Figure 2**). The aim was to distinguish labor initiation within a short period (TL to L) or at a similar gestational week (E+TL and L). ROC based on serum cortisol levels demonstrated similar performance in both groups (AUC 0.934-0.940), and cutoff values (21.46 μg/dL and 21.62 μg/dL), suggesting that serum cortisol levels were effective for the diagnosis of labor.

Labor diagnosis with a binary logistic regression model incorporated cortisol and age from the background information (**Table 2**). However, the age of patients in group TL was generally higher than that of group L. Therefore, we postulate that the inclusion of age might due to the convenience sampling bias. Besides, the correlation test demonstrated that age and cortisol levels were not related. The decision curves that provided a comprehensive representation of the strengths and weaknesses of classification methods implied a better risk and beneficial outcome for logistic regression analysis, possibly due to a better core algorithm (**Figure 3**).

Diagnosis of birth with machine learning methods such as Decision Tree (DT) or RF has been verified in previous studies (8, 17), incorporating multiple key factors and improving the prediction power of small datasets. Variables such as maternal age, gestation week, and cortisol level were used in common with previous studies, while parity and BMI before pregnancy were adopted only in this study (31). As shown in **Table 3**, the accuracy of the machine learning models was similar to that of the diagnosis based on the single indicator cortisol. With fewer variables used in the statistic model (cortisol only), the sensitivity and NPV were slightly higher (86.50% and 93.57%). In contrast, the 10-fold cross-validation of the SVM algorithm generated higher specificity and PPV (89.29% and 81.09%, **Table 3**). The similarity in confusion matrices and smooth calibration curves for the prediction algorithms suggested no overfitting for the machine learning models (**Figure 4**). Since labor initiation generally involves complicated physiological changes that are affected by many factors, processing of high-dimensional independent variables is preferred. More data are needed to further improve the performance of diagnosis, especially for the application of machine learning methods. Additionally, evaluation of the proposed management algorithms with larger population sizes and more variables is also an important work in the future.

Despite that cortisol has been used for the prediction of parturition in several studies, few mentioned the rapid change of cortisol between TL and L or tried to evaluate labor initiation with POCT (8, 17, 24). This cross-sectional study is the first to track serum cortisol levels onsite through different stages of pregnancy and distinguish TL from labor initiation. The sharp increase in serum cortisol level was likely caused by stress, pain, and part of the complicated physiological and psychological change of labor (18, 32). Based on the result of our study and the research of Miller et al. on salivary cortisol concentration through the course of labor, we postulate that serum cortisol might further increase in later phases of labor and be maintained at a high concentration for a short period postpartum (18). The rapid increase of serum cortisol levels, together with the age, gestation week, parity, and BMI of the patients could help to diagnose labor initiation effectively.

**Figure 5:**
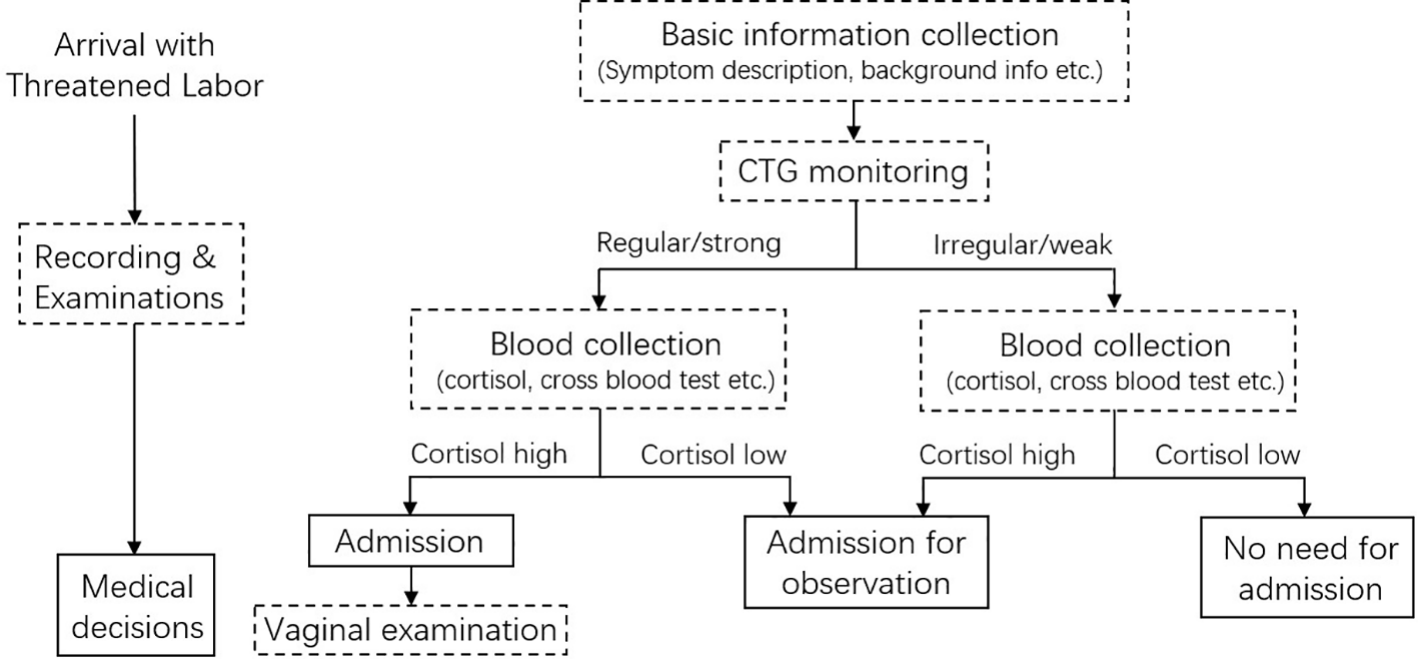
Workflow proposed for labor/threatened labor diagnosis with the inclusion of serum cortisol level.

Based on the practice of our hospital and this study, a workflow for TL/L diagnosis with the addition of serum cortisol tests was proposed (**Figure 5**). Since the clinical practice between hospitals and cultures varies, this workflow aims to guide if an additional parameter, cortisol concentration, is needed. Patients with TL symptoms could go through 3 types of examinations upon arrival: basic information collection, CTG monitoring, and blood tests. These operations could be conducted in parallel for time-saving. If both the contraction and cortisol levels are low, the admission might not be necessary. A strong contraction and high cortisol concentration might indicate an emergency. Serum cortisol tests can also be conducted after regular clinical examinations if the condition of patients is inconsistent. In special situations where the patient proceeds to the labor phase very quickly, or CTG and professionals are not available (i.e., in an ambulance), a quick, convenient cortisol test might help to make a preliminary decision.

The special design of this study includes the application of a POCT platform for onsite cortisol assessment. The ordinary examination procedures of the hospital were followed and the proposed methods could be transferred to clinical applications. The rapid increase of stress hormones was found from TL to labor initiation even with interpersonal variations between groups. Additionally, our study compared the serum cortisol levels of E, TL, and L group patients of similar gestation age, which was overlooked in previous studies (8, 18). Taking the design and detection tool of this study, future works should reveal the dynamic change of cortisol at different stages of labor and thereafter (33). In particular, it might provide insight into the prolonged latent phase or arrested active phase issues (34, 35).

A few limitations of our study should also be discussed to better interpret the results. Firstly, the sample size was effective in demonstrating the difference between TL and L groups, but relatively small for the application of machine learning algorithms. Following the standardized protocol, the tests and analysis need to be validated in more clinical sites, including a larger population with enough diversity, and more variables as potential influencing factors (22, 36). The generalizability across populations and the feasibility of the proposed machine learning method and workflow should be further verified. Secondly, due to the examination schedule of local patients, data from the T2 gestation period were missing. Fortunately, this information could be found in the study of Braithwaite et al., where pregnant women in the 3rd trimester had higher cortisol levels than in the 2nd trimester in both normal controls and depressed populations (37). Thirdly, variations might exist within the labor group, therefore future studies should evaluate different stages of labor.

In conclusion, we found that the E and TL patients demonstrated a similar cortisol concentration despite the differences in clinical symptoms. A sharp increase in serum cortisol level occurred within the relatively short period between TL and L, indicating a final countdown towards parturition. The serum cortisol level and basic information of patients could help to diagnose labor initiation with high accuracy. Using serum cortisol concentration as an additional biomarker for labor initiation may help decision-making and improve the healthcare of patients.

In the future, other investigations that apply cortisol measurements in the case of preterm birth could be attempted. Although preterm birth was not within the scope of this study, the non-invasive cortisol test in this study was quite convenient, making it easy to evaluate preterm birth, especially for those who visit the hospital in an emergency. The method of POCT, experimental design, and statistical analysis can be applied, and more comprehensive background information such as blood pressure and glucose level can be integrated. The physiological mechanisms underlying the observed rapid cortisol change should also be explored, including the testing of pCRH and ACTH, two upstream regulators of cortisol, at the same time point. This will further reveal the timely regulatory mechanism of hormones during TL and labor initiation, providing valuable insight into the utility of cortisol in clinical practice.

## Conclusions

This is the first cross-sectional study that focuses on the dynamic change of serum cortisol between TL and labor initiation, revealing the nearly doubled serum cortisol concentration between TL and L patients. A convenient POCT platform for serum cortisol was adopted, enabling the onsite rapid assessment of serum cortisol. With the same gestation week, the E and TL patients showed similar cortisol levels, although the physiological conditions were different. Conventional statistical analysis and machine learning algorithms were used for the distinguishment of threatened labor and labor with cortisol concentrations and multiple variables, reaching accuracy levels of 85.93%-89.79%. Thus, serum cortisol as an additional parameter might help to diagnose labor initiation and make medical decisions.

## Data availability statement

The raw data supporting the conclusions of this article will be made available by the authors, without undue reservation.

## Ethics statement

The studies involving humans were approved by the institutional review board of the Second Affiliated Hospital of Chongqing Medical University (clinical trial registration number: SAHCQMU 2021 No. 22.). The studies were conducted in accordance with the local legislation and institutional requirements. The participants provided their written informed consent to participate in this study.

## Author contributions

YC: Conceptualization, Formal analysis, Funding acquisition, Methodology, Project administration, Resources, Supervision, Writing – original draft, Writing – review & editing. HW: Data curation, Project administration, Software, Writing – original draft. DT: Data curation, Investigation, Writing – original draft. YW: Data curation, Investigation, Writing – original draft. ZS: Formal analysis, Investigation, Writing – original draft. YZ: Investigation, Validation, Writing – original draft. BZ: Investigation, Validation, Writing – original draft. BN: Data curation, Funding acquisition, Project administration, Resources, Software, Writing – original draft. XD: Conceptualization, Funding acquisition, Investigation, Resources, Writing – original draft, Writing – review & editing.
